# A Meta-Analysis of Randomized Controlled Trials on Acupuncture for Amblyopia

**DOI:** 10.1155/2013/648054

**Published:** 2013-04-30

**Authors:** Xingke Yan, Tiantian Zhu, Chongbing Ma, Anguo Liu, Lili Dong, Junyan Wang

**Affiliations:** Department of Acupuncture and Moxibustion, Gansu University of Traditional Chinese Medicine, Dingxi Dong Road No. 35, Lanzhou 730000, China

## Abstract

*Objective*. To assess the evidence of efficacy and safety of acupuncture for amblyopia and analyze the current situation of its clinical setting. *Methods*. We systemically searched Wanfang, Chongqing Weipu Database for Chinese Technical Periodicals (VIP), China National Knowledge Infrastructure (CNKI), and PubMed. Published randomized controlled trials (RCT) and controlled clinical trials (CCT) that evaluated the effect of acupuncture for amblyopia compared with conventional treatment were identified. The methodological quality of the included trials was assessed based on the Jadad scale. Data synthesis was facilitated using RevMan 5.1. *Results*. Fourteen trials involving 2662 participants satisfied the minimum criteria for meta-analysis. The evidence showed that the total effective rate of treatment within the group receiving acupuncture was higher than that in conventional group; there were statistically significant differences between groups (polled random effects model (RR) = 1.17, 95% confidence interval (1.11, 1.24), *Z* = 5.56, *P* < 0.00001). *Conclusion*. The total effective rate of acupuncture for amblyopia was significantly superior to conventional treatment, indicating that acupuncture was a promising treatment for amblyopia. However, due to the limited number of CCTs and RCTs, especially those of large sample size and multicenter randomized controlled studies that were quantitatively insufficient, we could not reach a completely affirmative conclusion until further studies of high quality are available.

## 1. Introduction

Amblyopia is a visual impairment (monocular or binocular) resulting from insufficient light stimulus entering eyes during congenital or critical period of visual development, depriving macula lutea of the opportunity of forming clear images, that is, so-called visual deprivation, and/or anisometropia leading to competition of clear image with fuzzy objects (binocular interaction abnormalities). Organic pathological changes cannot be found in general ophthalmologic examination, optical corrections ≤0.8 by cycloplegic retinoscopy correct [1]. Nowadays, available methods for amblyopia in clinical practice mainly include: patching, afterimage, synoptophore, visual physiological stimulation therapy, light therapy, He-Ne laser therapy, and L-dopa injection, all of which could achieve some therapeutic effect, but with a requirement for age. To older patients, the effect was not significant, and for children, the compliance was poor, therefore the effect was not satisfactory. In recent years, acupuncture as a popular treatment for amblyopia in clinical practice has various modalities. Its efficacy and safety have been evaluated in quite a few RCTs or CCTs. However, the quality of these reported RCTs or CCTs which investigated the efficacy of acupuncture on amblyopia have not been assessed systematically. Therefore, we conducted this meta-analysis (2001–2012) to provide the basic scientific evidence for clinical acupuncture practice.

## 2. Methods

Definitions of Randomized Controlled Trial (RCT) and Controlled Clinical Trial (CCT). We retrieved relevant RCTs and CCTs that are eligible for the inclusion according to the guidelines of International Cochrane Collaboration [2]. The clinical trial is defined as trial carried out on one or more patients, with concurrent comparison of two or more intervention measures. RCTs are those clinical studies in which subjects were assigned to different treatment groups using randomization allocation such as random number, computer-generated random sequences, tossing a coin, and draw lots. CCTs (quasi-randomized studies) are those that do not strictly adhere to randomized methods of allocation, for example, allocation by order of admission, hospital registration number, date of birth, day of the week, or some other method that is not truly random, or clinical trials which randomization methods could not be identified.

### 2.1. Data Sources and Search Strategy

The literature search was performed by using the following databases: Chongqing Weipu Database for Chinese Technical Periodicals (VIP), Wanfang, China National Knowledge Infrastructure (CNKI), and PubMed (January 2001 to March 2012). There were no restrictions regarding the language of publication. All databases were across-retrieved to avoid missing. The key words used for the search were amblyopia AND (acupuncture OR moxibustion OR electroacupuncture OR auriculotherapy OR auricular application pressure). In addition, those articles that could not be obtained were screened manually and independently in Jilin Provincial Library until 12 March 2012 by two authors.

### 2.2. Study Selection

Studies were eligible only if they met the following criteria: (1) the clinical trials on acupuncture treatment for amblyopia are published in biomedical journals (January 2001 to March 2012); (2) the sample size of patients >10; (3) randomized or quasi-randomized study designs which contained the control group; (4) treatment and control groups were allocated according to the number of affected eyes; (5) with clear diagnosis and effect criteria; and (6) comparing acupuncture treatment with other modalities.

Trials were excluded if any of the following were identified. (1) If the type of articles were animal experiments, review articles, case reports or expert experience reports, conference papers, or dissertations. (2) If studies are comparing two different forms of acupuncture or point selection and formulating prescription. Articles that investigated acupuncture as adjunctive therapy were excluded as well.

### 2.3. Data Extraction and Management

Data on study characteristics were abstracted independently by the two authors using a standardized collection form, which includes first author, year of the study, sample size, randomization, blinding, baseline characteristics, diagnosis and effect criteria, study selection (inclusion and exclusion criteria), interventions, main outcome assessments, follow-up time, withdrawal, adverse effects, and literature provenance. 

### 2.4. Methodological Quality Assessment

The methodological quality of retrieved articles was rated using the Jadad scale [3]. Any disagreements on study quality were resolved through reviewing the study and discussing the discrepancy. This scale consists of 4 criteria being: (1) random allocation of subjects and allocation concealment (two points for randomization scores. Studies that were described as randomized were given one point. A further point was given if the method of randomization was described and was appropriate, such as the use of a random numbers table); (2) except for intervention, the rest of measures being carried out in two groups were similar (between-group statistical comparisons); (3) blinding score (range 0~2). Studies that were described as double-blinded were given one point; a further point was given if blinding was appropriate, such as matched placebos; one point was deducted if blinding was inappropriate); and (4) if exclusion bias exists, in other words, whether there exist systematic differences in two groups on withdrawals (number and reasons of withdrawals for 0~1 point. If the number and reasons for withdrawals were described in the study, one point was given). The minimal and maximal scores for an included study were 1 and 5, respectively. We arbitrarily classified quality as high (score: 3–5) versus low (score: 0–2).

### 2.5. Data Synthesis and Analysis

Meta-analysis was conducted using RevMan 5.1 analyses software of the Cochrane Collaboration [4]. The method of analysis selected for this study was to calculate the risk ratio (RR) for each trial: and then conduct test for heterogeneity, the fixed effects models were used to combine effect size when better results of homogeneity presented (*P* > 0.05), on the contrary, using the random effects model. We selected RR as the effect size index and calculated the 95% confidence interval, where *P* < 0.05 was regarded as statistically significant.

## 3. Results

### 3.1. Study Characteristics

A total of 115 possible trials were identified, but only 14 satisfied the inclusion criteria, of which 101 were excluded in the screening process as they clearly did not meet the inclusion criteria. The English language literature search identified 10 articles, all of which were unavailable for this meta-analysis, while the Chinese literature search identified 105 articles, the title, abstract, and full text of which were reviewed and 14 full articles [5–18] were eligible for inclusion criteria. Because all articles were domestic periodicals, lingual bias existed in this study, and the published time was from 2001 to 2012. The methodological quality of each study is described in Table 1.

### 3.2. Quantitative Data Synthesis

**Figure 1:**
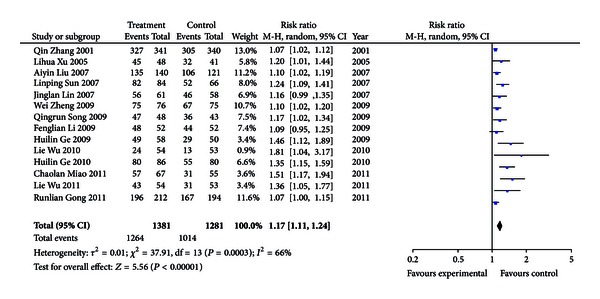
Meta-analysis of 14 trials (CI: confidence interval; *P*: *P*-value).

Through comparisons of the overall effectiveness of acupuncture treatment for amblyopia in 14 papers [5–18], the extent of heterogeneity in trials was *X* ^2^ = 37.91, *P* = 0.0003. The random-effects models were used to combine effect. RR = 1.17 showed that the beneficial influence of the experimental factors on disease was significant. The 95% confidence interval was 1.11, 1.24, indicating that acupuncture treatment for amblyopia was effective. The test results of combined effect were *Z* = 5.56, *P* < 0.00001, showing that it was statistically significant in two groups (Figure 1). Just as Figure 1 depicted, diamond falling on the right side of the vertical line showed that comparison of treatment group with the control group was of statistically significant differences.

### 3.3. Publication Bias Analysis

**Figure 2:**
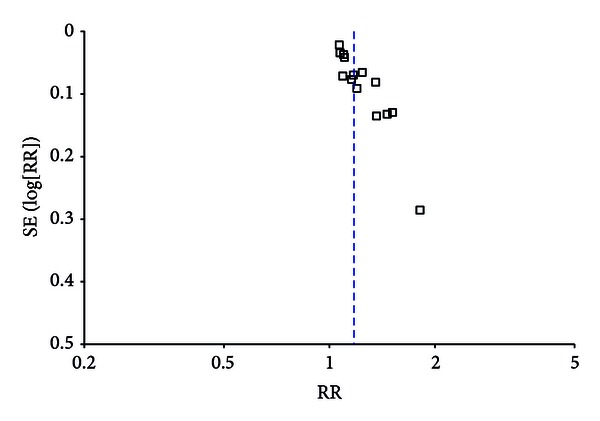
Funnel plot of publication bias analysis.

We assessed publication bias using the funnel plot on studies comparing acupuncture for amblyopia with conventional treatment. When there is no publication bias, points in funnel plot are almost matched. In this study points about the overall effect's comparison between groups presented asymmetry, suggesting the possibility of publication bias, which is shown in Figure 2. Although great efforts were made to retrieve all trials on the subject, we still could not exclude the possibility that studies with negative findings remain unpublished and parts of included studies were less relevant. In addition, language bias may exist because all included trials were published in Chinese. All in all, the total analysis displayed that publication bias, lingual bias, selection bias, and implementation bias in literatures included in the study may exist. 

## 4. Discussion

### 4.1. Summary of Main Results

This study performed a meta-analysis to look at the overall effect of included RCTs or CCTs contribute to acupuncture treatment for amblyopia. The evidence suggested effect of acupuncture treatment for amblyopia was superior to conventional treatment. However, through analysis of included 14 trials, we found several problems of study design, including: (1) inadequate method of the randomization; only 6 out of 14 papers [5, 8, 13, 15, 16, 18] described the specific randomization and others were without specific descriptions. This may have resulted in poor comparability of the treatment and control groups; (2) no reports of blinding: blinding was mainly employed to avoid additional variables triggered by the subjective expectation of assessor and subjects and to ensure that the result was reliable. Blinding was not used in 14 trials. Therefore implementation bias may exist; (3) unclear expression of baseline characteristics. Five out of 14 papers [5, 8, 9, 11, 16] only referred to the comparability, even without reports of baseline characteristics, resulting in poor comparability and selection bias. The above results caused the low quality scores of literatures and only 6 papers [4, 7, 14–17] scored ≥3 points, indicating that the quality of literatures was disappointing.

### 4.2. Strengths and Weaknesses

The main findings of this meta-analysis were that effect of acupuncture treatment for amblyopia was superior to conventional treatment. However, there are several limitations to this meta-analysis. One limitation is the limited number of CCTs and RCTs, especially those of high quality, large samples size, and multicenter randomized controlled trails. Other issues are that inclusion and exclusion criteria were not uniform, no detailed reports of blinding, follow-up and withdrawal, few high-quality studies, only published articles, lingual limitation, and selection and implementation bias. So the efficacy of acupuncture for amblyopia has not been proven beyond reasonable doubt, and further randomized controlled trials with better study methodology are needed.

## Figures and Tables

**Table 1:** The methodological quality of each study.

First author and date	Study design	Study selection			Methodology				Interventions		Baseline	Jadad score
		Diagnostic criteria	Inclusion/exclusion criteria	Effect criteria	Randomization	Blinding	Adverse effects	Reports of withdrawal and follow-up	Treatment group	Control group		
Zhang (2001) [5]	RCT	Refer to criteria of [19]	None	Refer to criteria of [19]	Random and concurrent control	No	No	Duration of follow-up: 3 years, no withdrawal	Mainly used acupuncture	Mainly used the traditional treatment	Adequate	3
Xu (2005) [6]	CCT	Refer to criteria of [19]	None	Refer to criteria of [19]	Only random words	No	No	Duration of follow-up: 3 years, no withdrawal	Mainly used acupuncture	Mainly used the traditional treatment	Adequate	2
Lin (2007) [7]	CCT	Refer to criteria of [18]	None	Refer to criteria of [18]	Only random words	No	No	None	Mainly used acupuncture	Mainly used the traditional treatment	Adequate	2
Liu (2007) [8]	RCT	Refer to criteria of [19]	None	Refer to criteria of [19]	Random and concurrent control	No	No	Duration of follow-up: 3 years, no withdrawal	Mainly used acupuncture	Mainly used the traditional treatment	Adequate	3
Sun (2007) [9]	CCT	Refer to criteria of [20]	None	Refer to criteria of [20]	Only random words	No	No	Duration of follow-up: 1 years, no withdrawal	Mainly used auricular seed-pressing therapy	Mainly used the traditional treatment	No report	1
Ge (2009) [10]	CCT	Refer to criteria of [20]	None	Refer to criteria of [20]	Only random words	No	No	Duration of follow-up: 3 years, no withdrawal	Mainly used acupuncture	Mainly used the traditional treatment	Adequate	2
Li (2009) [11]	CCT	Only reports but not date	None	Only report but not date	Only random words	No	No	Duration of follow-up: 18 months, no withdrawal	Mainly used auricular seed-pressing therapy	Mainly used the traditional treatment	No report	1
Song (2009) [12]	CCT	Refer to criteria of [20]	Only report the inclusion criteria	Refer to criteria of *Ophthalmologist Must-Read* [21]	Only random words	No	No	Duration of follow-up: 3 years, no withdrawal	Mainly used auricular seed-pressing therapy	Mainly used the traditional treatment	Adequate	2
Zheng (2009) [13]	CCT	Refer to criteria of [20]	None	Refer to criteria of [20]	Simple random	No	No	Mention of follow-up, no withdrawal	Mainly used acupuncture	Mainly used the traditional treatment	Adequate	2
Ge (2010) [14]	CCT	Refer to criteria of [20]	None	Refer to criteria of [20]	Only random words	No	No	Duration of follow-up: 3 years, no withdrawal	Mainly used acupuncture	Mainly used the traditional treatment	No report	1
Wu (2010) [15]	RCT	Refer to criteria of [20]	None	Refer to criteria of [20]	Block randomization and parallel control	No	No	None	Mainly used electrical plum-blossom needle	Mainly used the traditional treatment	Adequate	3
Gong (2011) [16]	RCT	Refer to criteria of [20]	Both	Refer to criteria of [20]	Table of random number	No	No	Duration of follow-up: 3 years, no withdrawal	Mainly used auricular seed-pressing therapy	Mainly used the traditional treatment	Adequate	3
Liao (2011) [17]	CCT	Refer to criteria of [20]	None	Refer to criteria of [20]	Only random words	No	No	Cases of withdrawal, no follow-up	Mainly used TCM heat-sensitive moxibustion	Mainly used the traditional treatment	Adequate	3
Wu (2011) [18]	RCT	Refer to criteria of [20]	Both	Refer to criteria of [20]	Block randomization and parallel control	No	No	Criteria of withdrawal, no follow-up	Mainly used electrical plum-blossom needle	Mainly used the traditional treatment	Adequate	4
